# Identification and Evaluation of Aromatic Volatile Compounds in 26 Cultivars and 8 Hybrids of *Freesia hybrida*

**DOI:** 10.3390/molecules26154482

**Published:** 2021-07-25

**Authors:** Shidan Weng, Xueqing Fu, Yu Gao, Tianlei Liu, Yi Sun, Dongqin Tang

**Affiliations:** 1 School of Design, Shanghai Jiao Tong University, Shanghai 200240, China; wsd8416@sjtu.edu.cn (S.W.); cathyluck@sjtu.edu.cn (X.F.); tianlei.liu@alumni.sjtu.edu.cn (T.L.); yaoyao32688@sina.com (Y.S.); 2 Instrumental Analysis Center, Shanghai Jiao Tong University, Shanghai 200240, China; shirlygao@sjtu.edu.cn

**Keywords:** *Freesia hybrida*, floral scent, VOCs, monoterpenes, heterosis

## Abstract

*Freesia hybrida* is a group of cultivars in the genus *Freesia* with a strong floral scent composed of diverse volatile organic compounds (VOCs). In this study, the VOCs of 34 *F. hybrida* were extracted and analyzed by headspace solid phase microextraction and gas chromatography mass spectrometry (HS-SPME-GC-MS). A total of 164 VOCs whose relative contents were higher than 0.05% were detected. The numbers of VOCs in all germplasms differed between 11 to 38, and the relative contents ranged from 32.39% to 94.28%, in which most germplasms were higher than 80%. Terpenoids, especially monoterpenes, were the crucial type of VOCs in most germplasms, of which linalool and D-limonene were the most frequently occurring. Principal component analysis (PCA) clearly separated samples based on whether linalool was the main component, and hierarchical clustering analysis (HCA) clustered samples into 4 groups according to the preponderant compounds linalool and (*E*)-β-ocimene. Comparison of parental species and hybrids showed heterosis in three hybrids, and the inherited and novel substances suggested that monoterpene played an important role in *F. hybrida* floral scent. This study established a foundation for the evaluation of *Freesia* genetic resources, breeding for the floral aroma and promoting commercial application.

## 1. Introduction

Volatile organic compounds are a series of small-molecular (below 300 Da) products of plants’ secondary metabolism [1]. So far, there have been over 1700 volatiles identified in 991 species [2]. VOCs are the medium by which plants to interact with other organisms and environment [3]. Plants attract specific pollinators and seed disseminators by emitting species-specific signals to help increase sexual reproduction efficiency [4,5,6,7]. In plant defense, VOCs emitted from damaged plants tissues can directly influence antagonistic visitor physiology by toxic or repelling compounds, or can indirectly attract herbivores’ natural enemies by specific compounds [1]. Volatiles can mediate plant-plant competition by allelopathy or chemical camouflage [3]. In addition, they can also perform a function in antimicrobial or antifungal defense [8,9].

Floral scent is a kind of well-known odorous VOCs released from floral tissues [10]. VOCs vary among petal, pistil, nectary, and calyx within the same flower and are diverse in different cultivars, varieties and genotypes [3], which decides the characteristic floral scent of one plant. There are many studies which have extracted and analyzed natural VOCs from ornamental flowers to explore the constitutions of their typical aroma. For instance, rose is famous for its signature sweet scent which primarily consists of geraniol, citronellol and nerol [11]. A total of 86 VOCs were identified in tea-scented cultivars [12]. These floral VOCs mostly belong to three types according to their metabolic pathways: terpenoids, benzenoids/phenylpropanoids and fatty acid derivatives [1]. There are also some products containing sulfur or nitrogen atoms originating from the metabolism of amino acids [2]. Terpenoids and fatty acid derivatives are the main classes within the rose scent. The fragrant oriental lily was found to emit predominantly monoterpenoids and benzenoids for its special scent [13]. Moreover, the living scent or essential oil extracted from flowers has health functions. The 3,5-di-methoxytoluene that effectively contributes to the rose scent has a strong sedative and relaxing effect [14].

*Freesia* (Iridaceae) originates from South Africa and has been artificially planted for 18 centuries. *Freesia hybrida*, the modern cultivar population, is formally regarded as the offspring of *Freesia corymbosa* and *Freesia leichtlinii* in the anfractuosity of breeding [15,16,17]. The fusion of diverse varieties’ genetic resources led to the complex genetic background of *F. hybrida*. It is an excellent bulb flower with various colors and an abundant aroma. Thus far, there have been some studies which have identified the VOCs of the scent of *F. hybrida*. The cultivar ‘Rijnvelds Golden Yellow’ was firstly found that the principal compound linalool accounted for 67.96% in 31 VOCs [18]. Subsequent studies also demonstrated that terpenoids, in which linalool was critical, dominated the scent of *F. hybrida* [19,20]. However, these studies usually focused on limited cultivars, leading to an unrepresentative summary of scent characteristics of plenty of freesia germplasms. More rapid cultivar innovation provides newer demand to summarize the recent situation of the floral scent of *F. hybrida,* which is valuable for the cut flower and essential oil industries. In addition, as the most important commercial breeding method, intervarietal crossing is generally applied to breeding new *F. hybrida* cultivars. Studying the VOCs of hybrids plays a fundamental role in breeding new fragrant cultivars. It has been found that the hybrid generations would synthesize some novel scent traits or evidently lost parental chemical compounds [21]. More data are still required to explore the changes which occur during cultivating and breeding.

Based on our previous persistent breeding and germplasms collection, we selected 34 germplasms to identify the VOCs in these fresh *F. hybrida* flowers. Compared to other methods, HS-SPME-GC-MS is rapid, solvent-free, inexpensive and accurate when used to identify VOCs. [22,23]. PCA and HCA were then performed to analyze the floral scent pattern of these germplasms. Moreover, we screened some hybrids to explore the inheritance and variation of VOCs between parents and hybrids. This study aims to comprehensively analyze the characteristic of *F. hybrida*, and form a theoretical basis for scent breeding in *F. hybrida*.

## 2. Results

### 2.1. Comparative Analysis and Evaluation of VOCs

**Figure 1:**
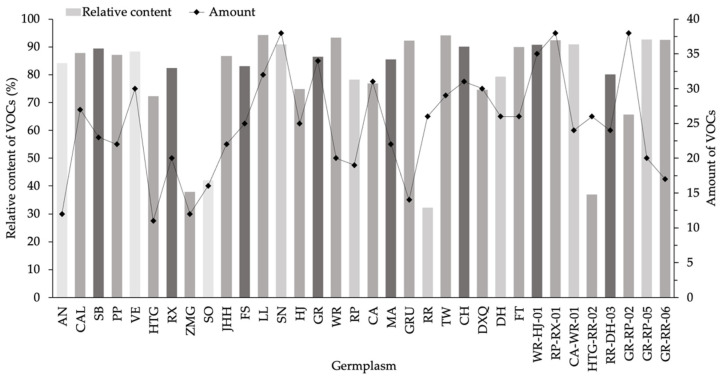
Relative content and numbers of VOCs in 34 *F. hybrida* germplasms.

Table 1 showed the information about 34 germplasms. A total of 164 VOCs whose relative content were higher than 0.05% were found in these germplasms (Table S1, Supplementary Material). The number of VOCs ranged from 11 only in HTG to 38 in three germplasms, including SN, RP-RX-01 and GR-RP-02 (Figure 1). Out of the 34 samples detected, the numbers of VOCs in 27 samples were found to exceed 20, of which 10 samples were above 30; other 7 samples contained less than 20 VOCs, of which HTG, ZMG, AN were as low as 11, 11 and 12. The percentages of VOCs’ relative contents accounted for 32.39% in RR to 94.28% in LL. A majority of germplasms contained high VOC contents, in which 23 samples were detected to have greater than 80%. The relative contents of all detected VOCs were present between 60% and 80% in 7 individual germplasms. Four samples, including SO, ZMG, RR and HTG-RR-02, contained VOCs with low percentages of less than 50%.

Numbers and relative contents of VOCs differed in tested freesia germplasms, and relative contents did not correspond to numbers. It was revealed that high VOC numbers did not mean high content for all germplasms. Typically, the germplasms with many VOCs were accompanied by high relative contents. However, the samples with large numbers of VOCs may contain low percentages (≤50%), such as RR and HTG-RR-02. Conversely, some germplasms emitted few VOCs and contained high VOCs percentages, including AN, HTG, GRU and GR-RR-06.

**Figure 2:**
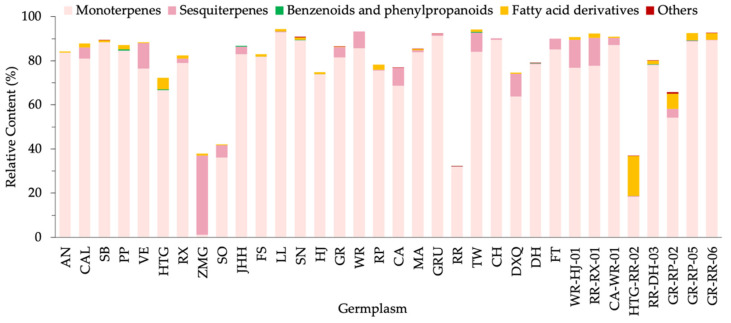
Relative content of different VOC types in 34 *F. hybrida* germplasms.

According to metabolic pathways, the VOCs could be classified into 127 terpenoids, 18 fatty acid derivatives, 9 benzenoids and phenylpropanoids and 10 other compounds (hydrocarbons, sulfur- and nitrogen-containing compounds) (Figure 2). Terpenoids, including 84 monoterpenes and 43 sesquiterpenes, dominated the floral scent in the majority of germplasms. In relative content terms, monoterpenes were also the most chief components. As shown in Figure 2, 33 samples contained more than half monoterpenes, except ZMG whose primary substance was sesquiterpenes. In most germplasms, fatty acid derivatives were observed in low contents, except for a high percentage in an individual germplasm, HTG-RR-02. Benzenoids and phenylpropanoids and other compounds were very low in most germplasms suggesting a negligible effect on *F. hybrida*.

Among detected VOCs, linalool, the compound with the highest frequency of occurrence, was found in all germplasms. Meanwhile, linalool was the highest percentage substance in 19 samples, with a range of relative abundance from 21.55% to 82.86%. In addition, d-limonene was also observed with the highest occurrence frequency of 34, followed by several monoterpenes, including myrcene, (*E*)-β-ocimene, α-terpineol, terpinolene and alloocimene, with an occurrence frequency of 33, 32, 28, 28 and 27, respectively. The other 73 compounds were only detected peculiarly in one certain sample, such as (*E*)-β-farnesene in CA, myrtenol in LL, *o*-methyl anisole in RR-DH-03, and so on.

### 2.2. Pattern of the Floral Composition in 34 Germplasms

In order to describe the composition patterns of floral VOCs in 34 germplasms, 52 aromatic compounds above 0.5% were used to visually evaluate the characters and variation in different samples by statistical methods.

**Figure 3:**
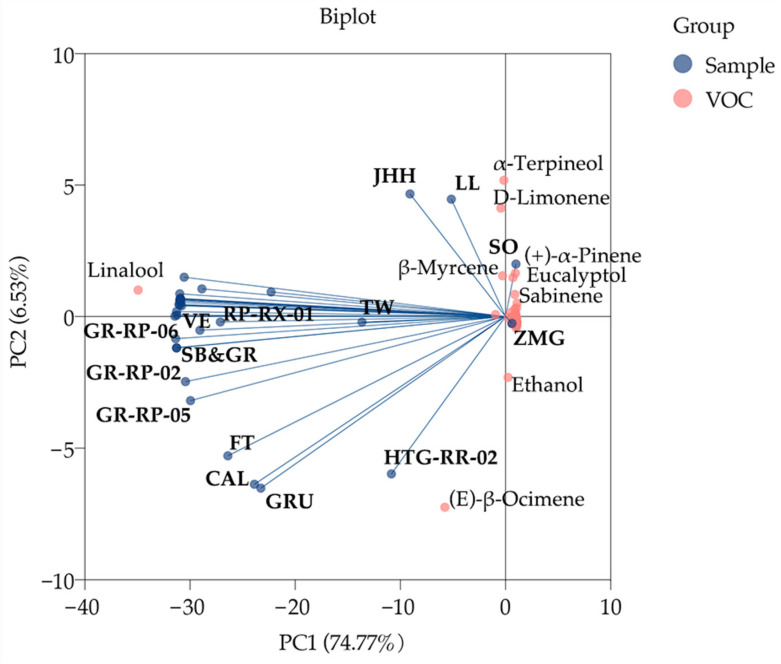
PCA biplot of VOCs in 34 *F. hybrida* germplasms.

PCA was performed to explore the preliminary classification of different samples and the important compounds correlated with them. The comparatively dispersive samples and evidently contributing VOCs were marked in the biplot (Figure 3). PC1 and PC2 explained 74.77% and 6.53% variances of compounds, respectively. The distribution of samples assumed two different trends. Most samples which linalool had a strong positive influence on were gathered; conversely the others dispersing far away from linalool were slightly affected. Of these linalool-affected samples, VE, GR-RP-06, SB, GR, GR-RP-02, GR-RP-05, SNFT, CAL, GRU and HTG-RR-02 were positively correlated with (*E*)-β-ocimene. SO (PC1, 0.988) and ZMG (PC1, 0.025), the samples that most positively correlated with PC1, were almost irrelevant to linalool, of which SO were highly affected by β-myrcene, eucalyptol, (+)-α-piene and sabinene. α-terpineol and d-limonene significantly impacted JHH and LL, but negatively impacted FT, CAL, GRU and HTG-RR-02. Ethanol had a positive influence on HTG-RR-02.

**Figure 4:**
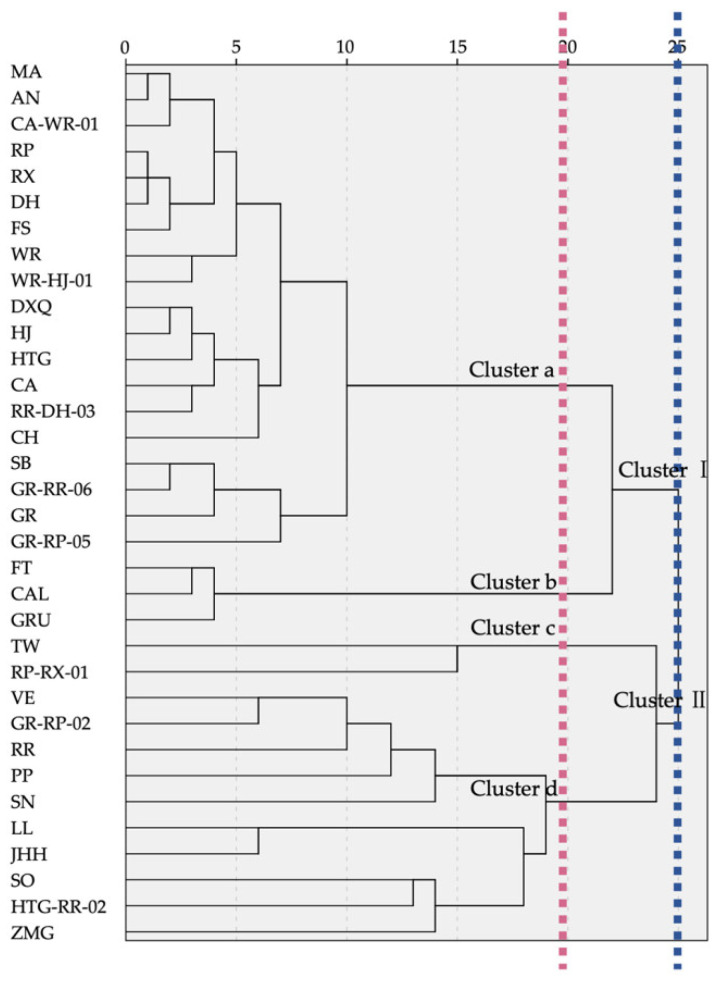
HCA dendrogram of VOCs of 34 *F. hybrida* germplasms.

As a result of HCA, the dendrogram showed that 34 samples were clustered distinctly into two groups by the proportion difference of VOCs when Euclidean distance was 25 (Figure 4). Cluster I contained 22 samples, in which there were noticeable preponderant compounds accounting for more than 55%, and the other components were observed in only a few percentages from 0.5% to 3%. The discussion of Cluster I was mainly about the predominant VOCs. When the distance was 20, Cluster I was divided into 2 subgroups based on the variation of dominant compounds. The 19 samples in Cluster a were representative of the only high percentage compound, linalool, and Cluster b, including 3 samples (FT, CAL, GRU), was prominently by linalool and (*E*)-β-ocimene. Cluster II contained 12 samples, with the composition pattern showing that the substances’ percentages were gradually decreasing from the chief substance less than 55% to low relative content VOCs. When the geometrical distance was 20, Cluster II was also classified into two subgroups according to composition differences. TW and RP-RX-01 were separated into group c due to their similar constituents and proportions of linalool, (*Z*)-β-ocimene, copaene, (*E*)-β-ocimene, cyperene, ethanol and rosefuran. In group d, the first high percentages were less than 45%, the second was less than 20%, the third was under 12% and the other constituents had a slight step-down in percentages. Within the 10 samples of group d, VE, GR-RP-2, RR, PP and SN were clustered together due to linalool being the highest percentage substance; SN was the only sample that contained nerol in high relative content. LL was closely identified with JHH for their semblable compositions, which contained, sequentially, α-terpineol, d-limonene, eucalyptol, β-myrcene, sabinene, linalool and so on. The last branch in group d contained SO, HTG-RR-02 and ZMG in low total relative contents, as well as various major compounds including (+)-α-pinene, ethanol and α-selinene, respectively.

### 2.3. Comparison of the VOCs of Hybrids and Parental Species in F. hybrida

In our previous breeding work, we collected 9 representative hybrids with different aroma qualities, which were offspring from the intervarietal crossing of 9 cultivars (WR, HJ, CA, RR, DH, HTG, RP, RX and GR). The comparison of VOC characteristics with hybrids and parental species was made to analyze the hybridization results of the *F. hybrida* flower scent.

**Figure 5:**
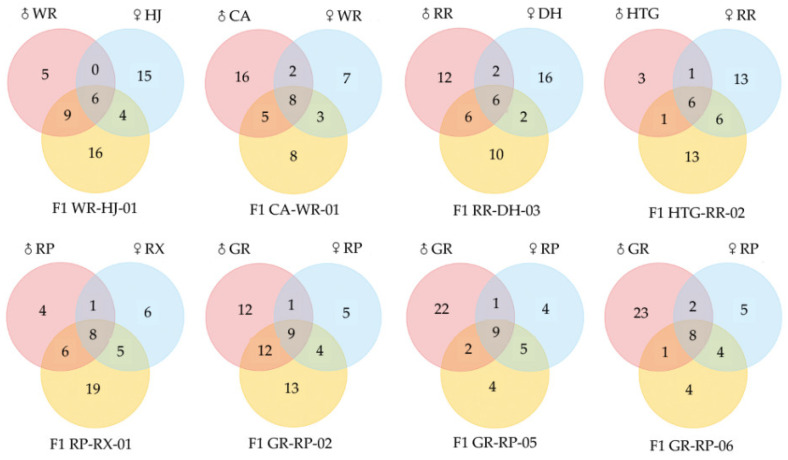
Venn diagrams of numbers of VOCs of 8 freesia hybrids with their parental species.

Figure 5 shows the quantitative comparison between parental species and hybrids. As a whole, 9 hybrids stably inherited a total of 16 compounds from both of their parents, and they contained steady VOCs with a range from 6 (in WR-HJ-01, RR-DH-03 and HTG-RR-02) to 9 (in GR-RP-05 and GR-RP-02), including linalool, (*E*)-β-ocimene, d-limonene, β-myrcene, cosmene and alloocimene and so on (Table 2). These VOCs were all monoterpenes; linalool, (*E*)-β-ocimene, d-limonene and β-myrcene were observed in each overlap between parents and hybrids, of which linalool showed high relative contents above 30% in most hybrids except HTG-RR-02 (6.03%). (*E*)-β-ocimene was higher than 6%, except in WR-HJ-01 (1.86%) and CA-WR-01 (0.89%). Conversely, d-limonene and β-myrcene emitted low levels under 1.50%. In addition, numbers of unique compounds in hybrids differed from 4 in GR-RP-05 and GR-RP-06 to 19 in RP-RX-01 when compared with their parents, such as cyperene in WR-HJ-01, sabinene in RR-DH-02, durenol in RP-RX-01 and so on. These specific substances in hybrids were almost always lower than 2.80%, excluding ethanol (17.92%) in HTG-RR-02.

**Figure 6:**
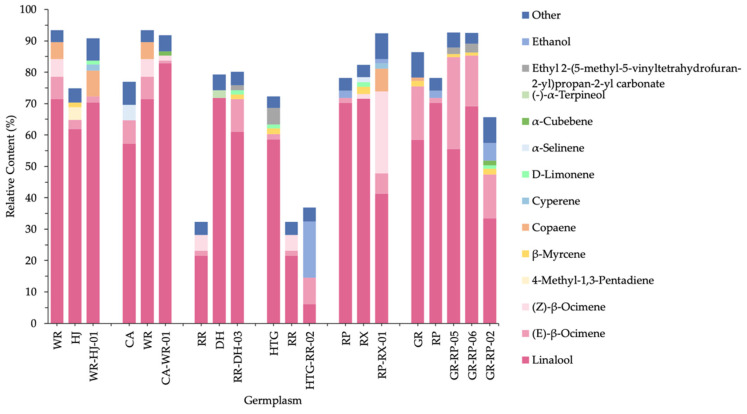
Relative content of total and important VOCs of parental species and hybrids.

Figure 6 showed the relative contents of total VOCs and some important compounds of parents and hybrids. It was observed that different parental species groups generated various results, and the relative contents in most hybrids were near to, or higher than, parental species. Firstly, in WR-HJ-01, CA-WR-01 and RR-DH-03, the VOC proportions of hybrids were closer to the parents, with the advantage of high VOCs relative content, mainly due to an increase in copaene, linalool and (*E*)-β-ocimene, respectively. Secondly, the percentage of HTG-RR-02 was observed in evident proximity to RR, the female parent with low VOC relative content, as a result of a decrease in linalool percentage and a substantial increase in ethanol percentage. Thirdly, heterosis was present in RP-RX-01, GR-RP-05 and GR-RP-06. This occurred in RP-RX-01 mainly because of the increase of ocimene. However, as the offspring of the same parents (GR and RP), GR-RP-02 contained the lowest VOCs, even less than parents, which was also observed via linalool relative content decrease and ethanol content increase.

## 3. Discussion

Richness of VOCs in numbers and relative contents reflects the diversity of different resources. We obtained the most floral scent compounds from 34 *F. hybrida* germplasms so far. There are more VOCs detected in *F. hybrida* than other species, such as *Prunus mume*, which emitted 31 VOCs in 8 cultivars [24]. As the most abundant floral scent volatiles, terpenoids widely exist in many scented plants, including β-ionone and linalool in *Osmanthus fragrans* [25]. In most detected *F. hybrida*, monoterpenes contribute the majority of VOCs. There are also sesquiterpenes serving as the chief compounds in some freesia cultivars, including ZMG and ‘Rose Marie’ [26]. Similar results were reported that fatty acid derivatives may play a certain role in *Freesia* fragrance [21]; and some wild *Dianthus* also emit them as principal VOCs [27]. Though benzenoids and phenylpropanoids have no evident effects on *F. hybrida*, they are the primary VOCs in *Petunia axillaris* [28], *Antirrhinum majus* [29], and *Hedychium coronarium* [30]. In addition, these results are nearly consistent with the result that linalool is the predominant VOC with a range from 33.60% to 82.51% in most *F. hybrida* in previous reports [18,26,31]. Linalool is also a primary volatile substance in many species, which occurs in more than half of the families of seed plants [2]. Other compounds like terpinolene and alloocimene, the important volatiles firstly reported in *F. hybrida*, contribute specificity to the germplasm.

The scent of VOCs is closely related to their composition. It was demonstrated that the number of VOCs had little effect on a sensory evaluation survey, suggesting that the floral odor was predominated by the principal volatile compound and its content [32]. Lower content and higher odor threshold values lead to weaker perceptions of floral scent [33]. Among the germplasms dominated by linalool, RR is almost without fragrance, due to the low content proportion of linalool compared to the other germplasms. Moreover, the principal VOCs of LL and SO are α-terpineol and (+)-α-pinene, whose odor value thresholds are 0.01–110 mg/m^3^ and 0.0053–23 mg/m^3^, respectively. These substances were observed in low proportions and their odor value thresholds were much higher than linalool (0.0004–6 mg/m^3^), so their scent was imperceptible [34].

Based on PCA and HCA, there are various composition patterns of VOCs of F. *hybrida*. Undoubtedly, linalool significantly contributes the floral scent of most germplasms. Interestingly, there are also other types of VOC composition observed in *F. hybrida*, including those which are dominated by (*E*)-β-ocimene, predominant in (*Z*)-β-ocimene, linalool and copaene simultaneously, and predominant in α-terpineol, d-limonene and eucalyptol and other substances, respectively. (*Z*)-β-ocimene are even higher than linalool in TW. These substances were first observed as the major components in *F. hybrida*. As an inherited trait, the similar VOC compositions in the same group may possess a close evolutionary relationship [14,35], so the high scent similarity of LL and JHH may be due to close ancestors. Having various major VOC compositions is beneficial to the diversity of floral scent. According to scent classification, 29 *Tulipa* cultivars were classified into 9 groups, including anise, citrus, fruity and six other scented groups [36]. These above findings will enhance the understanding of the floral scent of *F. hybrida* and are helpful to exploit the developmental potential of different *F. hybrida* scent types in future.

Intervarietal crossing is frequently used as an important method to breed new varieties for important commercial flowers including *Freesia*. As a showy trait, excellent floral scent is also considered as a key breeding purpose in *Freesia*. The stable inheritance of some substances is evidently influenced by the level and activity of enzymes [37,38]. All of these monoterpenes are synthesized by the MEP pathway, suggesting that MEP is the active and descendible metabolic pathway in producing VOCs in *F. hybrida*. Coincidentally, the novel substances generated in hybrids are almost monoterpenes as well, which implies the differential expression of downstream synthetases in the MEP pathway. This study even found that the presence of a high-percentage fatty acid derivative in a *F. hybrida* hybrid, which was different from its parental species [21], indicating an absolute difference generating from another biochemical pathway [39].

Furthermore, higher VOC contents and abundant combinations are beneficial in breeding novel varieties with stronger fragrances and different scent types. In this study, we found that some hybrids generated higher relative contents of specific volatiles. Compared to the male parent WR, the percentage of copaene that smelt peppery was observed to be raised in hybrid WR-HJ-01. Hybrids, like WR-HJ-01 that are superior to one parental species, can serve in unidirectional backcrossing to enhance this special scent performance [39]. Additionally, there is heterosis in the offspring of GR and RP, such that the 05 and 06 hybrids individually obtained higher VOC content; while the 02 hybrid conversely emanated a low floral scent content but generated many novel compounds that existed in neither parental species, which may be a result of transgressive inheritance [40]. These novel VOCs can provide the potential of forming different floral scent compositions when breeding new varieties. Meanwhile, it may also be due to the susceptibility to the environment of floral scent [41]. In consequence, intervarietal crossing can keep the beneficial floral scent traits of parental species, and an increase in certain VOCs can enhance aromatic odor. The newly-generated substances can enrich the diversity of *Freesia* scents and provide different flavors for the breeding of new cultivars.

## 4. Materials and Methods

### 4.1. Plant Materials

**Figure 7:**
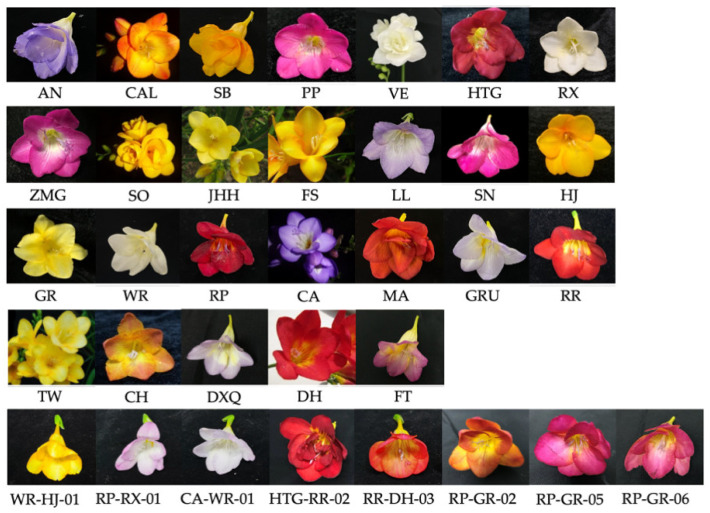
Flowers of 34 *F. hybrida* germplasms.

Thirty-four *F. hybrida* germplasms, including 26 cultivars and 8 interspecific hybrids, were cultivated under the same conditions in the standard polyhouse of Modern Agricultural Engineering Training Center of Shanghai Jiao Tong University (Figure 7, Table 1). Fresh flower branches were cut during 9:00~10:00 am. According to previous results, the fully open flower stage emitted the most abundant fragrant VOCs. Hence, we selected petals in the full open flower period to study the floral scent characters of all germplasms (Figure S1, Supplementary Material).

### 4.2. HS-SPME-GC-MS

Fresh petal samples (1.0 g) were weighted into a capped solid-phase microextraction vial (20 mL). Headspace sampling was done using 50 μm DVB/PDMS/CAR fiber, and the SPME fiber was exposed to the headspace of the sample for analyte extraction for 30 min at 40 °C by the CombiPAL autosampler (CTC Analytics, Zwingen, Switzerland).

GC-MS analyses were performed on a gas chromatograph-mass spectrometer (GC-MS 7890B-5977B, Agilent, Santa Clara, CA, USA) coupled with a DB-Wax column (30 m × 0.25 mm × 0.25 µm). The SPME fiber was desorbed into the GC injection port at 260 °C for 5 min in splitless mode. Helium (99.999%) was used as the carrier gas at a constant flow rate of 1.0 mL/min. The oven temperature was programmed from 40 °C for 5 min to 220 °C at 5 °C/min, and increased to 250 °C for 2.5 min at 20 °C/min. MS were operated in electron impact (EI) mode at 70 eV within the mass range 20–400 amu. The transfer line, ionization source and quadrupole were thermostated at 260 °C, 230 °C and 150 °C, respectively.

### 4.3. Data Analysis

Qualitative analysis was based on the comparison of experimental spectra with the National Institute of Standards and Technology (NIST) 2014 database and with published data [42]. Further identification was confirmed by retention index (RI) and NIST library similarity index. RI was calculated according to the data of a n-alkane (C7-C40) mixture standards. When standard RI was not available, NIST library similarity index (SI) was using as an auxiliary qualitative index. The baseline of compounds with a minimal relative content may cause a significant impact to mass spectra information, and lead to low SI (≤75%). The relative content data were calculated by peak area normalization measurement and by the following Equation (1):

(1) $$\mathrm{Relative}\;\mathrm{content}\;(\%)\;=\;100\;\times \;\frac{\mathrm{peak}\;\mathrm{area}\;\mathrm{of}\;\mathrm{compound}}{\mathrm{total}\;\mathrm{peak}\;\mathrm{area}\;}$$

The compounds (≤0.05%) were excluded for few effects to aromatic profile. Statistical analysis was performed by IBM SPSS 14.0. *Z*-normalization was carried out before PCA. Euclidean distance was used as proximity measurement for HCA.

## 5. Conclusions

*F. hybrida* is one kind of famous fragrant flower. This study collected by far the most germplasms to explore the aromatic VOCs of *F. hybrida*. 164 VOCs whose relative contents were higher than 0.05% were detected in 34 germplasms, and most germplasms contained many VOCs, as well as a high relative content of VOCs. We focused on the 52 compounds above 0.5% and found that linalool was the most predominant VOC in many germplasms, and also observed that D-limonene, (*E*)-β-ocimene and other monoterpenes were important in *Freesia*. PCA and HCA showed the separation of germplasms between linalool-dominated and other 3 compounds’ constituent patterns. Similar VOC compositions showed the close evolutionary relationship among germplasms. Abundant stably-inherited and newly-created compounds demonstrated that monoterpenes were the most important VOCs of the floral scent of *F. hybrida*. Besides the samples dominated by linalool, we anticipate obtaining more fragrance types with novel VOCs composition by intervarietal crossing. These findings establish the overall conception of the floral scent of *F. hybrida* from different aspects, providing a reference of directed breeding for enriching the pleasant fragrance of *F. hybrida*.

## Figures and Tables

**Table 1:** Sample of 34 *F. hybrida* germplasms.

No.	Code	Germplasm Name	Source ^1^	Sample Type
1	AN	‘Ancona’	a	Cultivar
2	CAL	‘Calvados’	a	Cultivar
3	SB	‘Summer Beach’	a	Cultivar
4	PP	‘Pink Passion’	a	Cultivar
5	VE	‘Versailles’	a	Cultivar
6	HTG	‘SN ^2^ Hongtaige’	b	Cultivar
7	RX	‘SN Ruxiang’	b	Cultivar
8	ZMG	‘SN Zimeigui’	b	Cultivar
9	SO	‘Soleil’	a	Cultivar
10	JHH	‘SN Jinhuanghou’	b	Cultivar
11	FS	‘Fragrant Sunburst’	a	Cultivar
12	LL	‘Lovely Lavander’	a	Cultivar
13	SN	‘Snoozy’	a	Cultivar
14	HJ	‘SN Huangjin’	b	Cultivar
15	GR	‘Gold River’	a	Cultivar
16	WR	‘White River’	a	Cultivar
17	RP	‘Red Passion’	a	Cultivar
18	CA	‘Castor’	a	Cultivar
19	MA	‘Mandarine’	a	Cultivar
20	GRU	‘Grumpy’	a	Cultivar
21	RR	‘Red River’	a	Cultivar
22	TW	‘Tweety’	a	Cultivar
23	CH	‘SN Chenghuang’	b	Cultivar
24	DXQ	‘SN Danxueqing’	b	Cultivar
25	DH	‘SN Dahong’	b	Cultivar
26	FT	‘SN Feitao’	b	Cultivar
27	WR-HJ-01	‘White River × SN Huangjin—01’	b	Hybrid
28	RP-RX-01	‘Red Passion × SN Ruxiang—01’	b	Hybrid
29	CA-WR-01	‘Castor × White River—01’	b	Hybrid
30	HTG-RR-02	‘SN Hongtaige × Red River—01’	b	Hybrid
31	RR-DH-03	‘Red River × SN Dahong—03’	b	Hybrid
32	GR-RP-02	‘Gold River × Red Passion—02’	b	Hybrid
33	GR-RP-05	‘Gold River × Red Passion—05’	b	Hybrid
34	GR-RR-06	‘Gold River × Red Passion—06’	b	Hybrid

^1^ a indicates the sample originated from Van den bos Co., Ltd. of Netherland; b indicates the sample originated from Shanghai Jiao Tong University. ^2^ SN indicates cultivars bred by Shanghai Jiao Tong University.

**Table 2:** Frequency of the VOCs of freesia hybrids inherited from both parental species.

No.	Name	Frequency
1	Linalool	6
2	β-Myrcene	6
3	d-Limonene	6
4	(E)-β-Ocimene	6
5	α-Terpineol	4
6	(+)-α-Pinene	3
7	Alloocimene	3
8	Terpinolene	2
9	(*E*)-Dehydroxylinalool Oxide	2
10	Rosefuran	1
11	(*E*)-Linalool Oxide (Pyranoid)	1
12	Ethanol	1
13	Cosmene	1
14	Dihydro-β-Ionone	1
15	β-Elemene	1
16	Selina-4(15),7(11)-Diene	1

## Supplementary Materials

The following are available online, Figure S1: Flowers of *F. hybrida* ‘SN Jinhuanghou’ in four developmental stages, Table S1: Relative contents (>0.05%) of floral scent compounds in 34 *F. hybrida* germplasms.
