# Supplementary material for: Identification and Evaluation of Aromatic Volatile Compounds in 26 Cultivars and 8 Hybrids of Freesia hybrida
Source: Molecules. 2021 Jul 25;26(15):4482. doi: 10.3390/molecules26154482 (PMC8347352; doi:10.3390/molecules26154482)
Supplement: Supplementary file 1 [file molecules-26-04482-s001.zip › molecules-1288747-supplementary.pdf]

SUPPLEMENTARY INFO

Identification and evaluation of aromatic volatile compounds in 26 cultivars and 8 hybrids of *Freesia hybrida*

Shidan Weng [1], Xueqing Fu [1], Yu Gao [2], Tianlei Liu [1], Yi sun [1] and Dongqin Tang [1,*]

[1] School of Design, Shanghai Jiao Tong University, Shanghai 200240, China

[2] Instrumental Analysis Center, Shanghai Jiao Tong University, Shanghai 200240, China

Correspondence to:

Dongqin Tang
School of Design, Shanghai Jiao Tong University, Shanghai 200240, China
E-mail: dqtang@sjtu.edu.cn;
Tel.: +86-21-34205730

Supplementary Figure

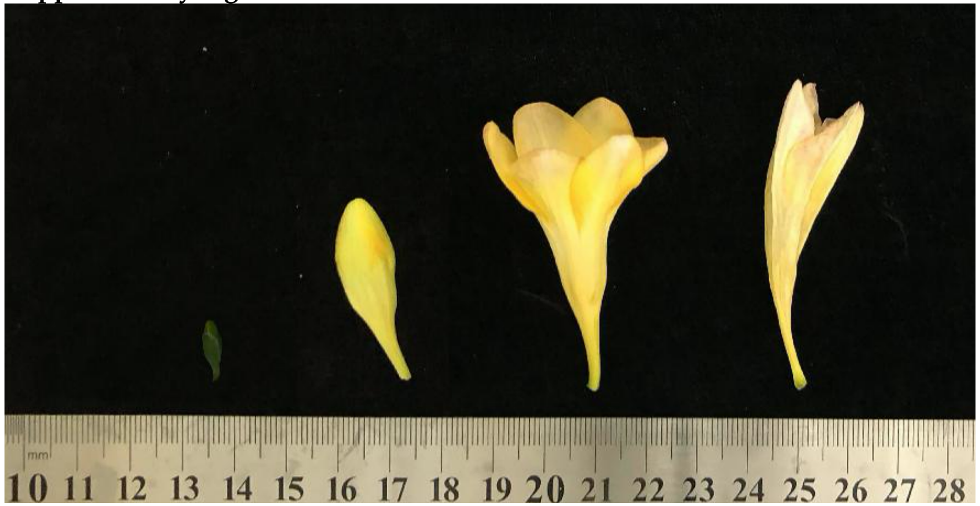

Figure S1. Flowers of *F. hybrida* 'SN Jinhuanghou' in four developmental stages (From left to light: green bud, bud with full color, full open flower, wilting flower).

Supplementary Table

Table S1. Relative content (>0.05%) of VOCs in 34 *F. hybrida* germplasms.

**Compounds**	**CAS**	RI	**SI (%)**	**Relative content (%)**																		
				**AN**	**CAL**	**SB**	**PP**	**VE**	**HTG**	**RX**	**ZMG**	**SO**	**JHH**	**FS**	**LL**	**SN**	**HJ**	**GR**	**WR**	**RP**	**CA**	**MA**
Ethyl DL-Leucate	10348-47-7	N/A	9	-	-	-	-	-	-	-	-	-	-	-	-	0.15	-	-	-	-	-	-
Vinyl Formate	692-45-5	N/A	5	-	-	-	-	-	-	-	-	-	-	-	-	0.20	-	-	-	-	-	-
Acetaldehyde	75-07-0	N/A	83	-	-	-	-	-	-	-	-	-	-	-	-	-	-	-	-	-	-	-
4-Methyl-1,3-Pentadiene	926-56-7	N/A	94	-	-	-	-	-	-	-	-	-	-	-	-	-	4.15	-	-	-	-	-
3-Methylfuran	930-27-8	941.82	91	0.20	-	-	-	0.09	-	-	-	-	-	0.07	-	0.24	-	-	-	-	-	-
2-Methyl Furan	534-22-5	941.97	91	-	-	-	-	-	-	-	-	-	-	-	-	-	-	-	-	-	-	0.05
Methyl ethyl ketone	78-93-3	943.87	86	-	-	0.06	-	-	-	-	-	-	-	-	-	-	0.11	-	-	-	-	-
Ethyl Acetate	141-78-6	N/A	91	-	-	-	0.21	-	-	0.17	-	-	-	-	-	-	-	-	-	-	-	-
Nonane	111-84-2	N/A	86	-	-	-	-	-	-	-	-	-	-	-	-	-	-	-	-	-	-	-
Ethanol	64-17-5	959.32	91	-	1.27	-	0.79	-	-	0.65	0.86	-	-	-	-	-	-	-	-	2.34	-	0.53
α-Thujene	2867-05-2	1021.30	94	-	-	-	0.24	-	-	-	-	-	1.60	-	-	-	-	-	-	-	-	-
Toluene	108-88-3	1035.70	74	-	-	-	-	-	-	-	-	-	-	-	-	-	0.11	-	-	-	-	-
L-α-Pinene	7785-26-4	N/A	97	-	0.24	0.20	0.95	1.52	-	-	-	-	-	-	4.35	-	-	-	-	-	0.15	-
Cyclofenchene	488-97-1	N/A	97	-	-	-	-	-	-	-	-	-	-	-	-	-	-	-	-	-	-	0.06
(+)-α-Pinene	7785-70-8	1015.90	97	-	0.24	-	-	-	0.69	0.40	-	14.56	5.65	0.36	-	0.74	0.74	0.1	-	0.17	-	-
Camphene	79-92-5	1056.10	97	-	-	-	-	-	-	0.10	-	9.4	0.35	-	-	-	-	-	-	-	-	-
2-Methyl Butyronitrile	18936-17-9	1085.70	59	-	-	-	-	-	-	-	-	-	-	-	-	-	-	-	-	-	0.05	-
Undecane	1120-21-4	1091.10	95	-	-	0.08	-	-	-	-	-	-	-	-	-	-	-	0.07	-	-	-	0.19
(-)-β-Pinene	18172-67-3	1091.50	94	-	-	-	-	-	-	-	-	-	-	0.12	-	-	-	-	-	-	-	-
(-)-Camphene	5794-04-7	N/A	97	-	-	-	-	-	-	-	-	-	-	-	0.32	-	0.26	-	-	-	-	-
1,4-Butanediol Diacrylate	1070-70-8	N/A	56	-	-	-	-	-	-	-	-	-	-	-	-	-	-	-	-	-	-	-
Phytane	638-36-8	N/A	72	-	-	-	-	-	-	-	-	-	-	-	-	-	-	-	-	-	-	-
2-Methyl Decane	6975-98-0	N/A	50	-	-	-	-	-	-	-	-	-	-	-	-	-	-	-	-	-	-	-
β-Pinene	127-91-3	N/A	90	-	-	-	-	0.32	-	-	-	3.57	1.42	-	1.36	0.18	0.13	0.07	-	-	-	-
β-Thujene	28634-89-1	1105.90	94	-	-	-	-	-	-	0.13	-	-	-	-	-	0.50	-	-	-	-	-	-
Sabinene	3387-41-5	1107.20	96	-	-	0.09	0.45	1.44	-	-	-	0.55	5.32	0.43	4.42	-	-	-	-	-	-	-
α-Phellandrene	99-83-2	1142.70	91	-	-	-	-	-	-	-	-	-	-	-	2.56	0.06	-	0.05	-	-	-	-
Agmatine	306-60-5	1143.40	17	-	-	-	-	-	-	-	-	-	-	-	-	-	-	-	-	-	-	-
β-Myrcene	123-35-3	1148.80	93	2.48	1.15	1.88	6.46	3.71	1.88	2.43	-	0.54	7.47	3.52	8.07	3.44	1.48	1.67	0.66	0.96	0.47	0.85

(+)-2-Carene	4497-92-1	N/A	97	-	-	-	0.77	0.37	-	-	-	-	0.82	-	0.76	-	-	-	-	-	0.08	-
1-Methylene-2-Methyl-3-Isopropenylcyclopentane	56710-83-9	N/A	91	-	-	-	-	-	-	-	-	-	-	-	-	-	-	-	-	-	-	-
α-Terpinene	99-86-5	1156.50	97	-	-	0.08	-	-	-	-	-	-	-	-	-	-	-	-	-	-	-	-
2,3-Dihydro-1,8-Cineole	92760-25-3	1182.70	72	-	-	-	-	-	-	-	-	-	-	-	-	-	-	-	-	-	-	-
Eucalyptol	470-82-6	1191.10	90	-	0.23	-	0.87	1.60	-	-	-	-	7.11	1.20	8.94	1.01	-	0.12	-	-	-	-
Dipentene	138-86-3	1193.20	94	-	-	-	-	-	-	-	-	-	-	-	-	-	-	-	-	0.08	0.08	-
(E)-dehydroxylinalool oxide	54750-70-8	N/A	97	0.11	-	0.19	-	8.28	-	0.11	-	-	-	-	-	-	0.05	0.11	-	0.11	-	-
D-Limonene	5989-27-5	1176.10	97	0.68	0.60	0.75	11.70	5.86	1.14	1.43	0.47	6.24	18.14	2.36	16.28	2.56	0.70	0.88	0.17	0.5	0.63	0.35
(Z)-Dehydroxylinalool Oxide	54750-69-5	1198.90	59	-	-	-	-	-	-	-	-	-	-	0.05	-	-	-	-	-	-	-	-
β-Terpinene	99-84-3	1219.70	91	-	-	-	-	-	-	-	-	-	-	-	-	-	-	0.33	-	-	-	-
(E)-β-Ocimene	13877-91-3	1241.00	94	0.55	41.43	18.44	3.11	17.36	1.72	0.51	-	-	0.97	0.55	0.40	0.75	2.92	17.08	7.07	1.59	7.46	0.17
3,7,7-trimethyl-1,3,5-cycloheptatriene	3479-89-8	N/A	81	-	-	-	-	-	-	-	-	-	-	-	-	0.20	-	-	-	-	-	-
(1S,3S)-(E)-4-Carene	5208-50-4	1261.40	91	-	-	-	-	-	-	-	-	-	-	-	-	-	-	-	-	-	-	-
(+)-3-Carene	498-15-7	N/A	94	-	-	-	-	-	-	-	-	-	-	-	-	-	-	-	-	-	0.17	-
α-Pinene	80-56-8	N/A	95	-	-	-	-	-	-	-	-	-	-	-	-	-	-	0.42	-	-	-	-
β-phellandrene	555-10-2	1184.30	95	-	0.09	0.09	0.30	0.23	-	-	-	-	-	0.19	0.68	0.26	0.08	0.11	-	-	0.06	-
γ-Terpinene	99-85-4	1226.90	97	-	-	0.06	1.38	0.47	-	0.13	-	-	1.49	0.17	1.26	0.42	0.11	-	-	-	-	-
(Z)-β-Ocimene	3338-55-4	1239.60	95	1.89	-	-	-	-	-	1.40	-	-	0.72	1.60	0.95	5.61	-	-	5.63	-	-	1.68
α-Ocimene	502-99-8	1243.50	95	-	-	-	-	-	-	-	-	-	-	-	-	-	-	-	-	-	-	-
Cyclopropane, 1,1-dimethyl-2-(3-methyl-1,3-butadien-1-yl)-	68998-21-0	N/A	93	-	0.15	0.09	-	-	-	-	-	-	-	-	-	-	-	-	-	-	-	-
Bicyclo[3.1.1]hept-2-ene,3	4889-83-2	1254.60	94	-	-	-	-	-	-	-	-	-	-	-	-	-	-	-	-	-	-	-
o-Cymene	527-84-4	1256.90	94	-	-	-	0.35	-	-	-	-	-	0.28	0.12	-	-	-	-	-	-	-	-
Isoterpinolene	586-63-0	1259.40	90	-	-	-	-	-	-	-	-	-	-	-	0.05	-	-	-	-	-	-	-
m-Cymene	535-77-3	1263.50	93	-	-	-	-	-	-	-	-	-	-	-	0.25	-	0.05	0.11	-	-	-	-
Terpinolene	586-62-9	1266.20	97	0.13	0.09	0.13	1.28	0.70	0.33	0.24	-	0.27	2.13	0.17	3.14	-	0.35	0.87	-	0.17	0.38	0.12
(+)-4-Carene	29050-33-7	1267.40	96	-	-	-	-	-	-	0.24	-	-	-	-	-	0.87	-	-	-	-	-	-
p-Cymene	99-87-6	1270.40	91	-	-	-	-	-	-	-	-	-	0.28	-	-	-	-	-	-	-	0.12	-
δ-3-carene	13466-78-9	N/A	91	-	-	-	-	-	-	-	-	-	-	-	-	-	-	-	-	-	-	-

2-Ethyl-1,4-Dimethyl Benzene	1758-88-9	N/A	60	-	-	-	-	-	-	-	-	-	-	-	-	-	-	-	-	-	-	-
2-Carene	554-61-0	N/A	96	-	-	-	-	-	-	-	-	-	-	-	-	-	-	-	-	0.06	-	-
Tridecane	629-50-5	1295.20	95	-	-	-	-	-	-	-	-	-	-	-	-	0.05	-	-	-	-	-	-
Oxalic acid, allyl pentyl ester	1000309-23-2	1332.10	64	-	-	-	-	-	-	-	-	-	-	-	-	-	-	-	-	-	-	-
Methyl Heptenone	110-93-0	1336.30	92	-	-	-	-	-	-	-	-	-	-	0.05	-	0.11	-	-	-	-	-	-
Pentyl Propyl Ether	18641-82-2	N/A	78	-	-	-	-	-	-	-	-	-	-	-	0.53	-	-	0.07	-	-	-	-
Alloocimene	673-84-7	1372.60	94	0.17	1.01	0.50	0.07	-	-	-	-	-	-	0.20	0.05	0.47	0.06	0.34	0.14	0.06	0.09	0.12
1,5,5,6-Tetramethylcyclohexa-1,3-Diene	514-94-3	1375.90	94	-	-	-	-	-	-	-	-	-	-	-	-	-	-	-	-	-	-	-
3,4-Dimethyl-2,4,6-Octatriene	57396-75-5	1378.60	87	-	-	-	-	-	-	-	-	-	-	-	-	-	-	-	-	-	-	-
1,2,3,4,5-Pentamethylcyclopentadiene	4045-44-7	1381.30	97	-	-	-	-	-	-	-	-	-	-	-	-	-	-	-	-	-	-	-
p-Mentha-1,3,8-Triene	18368-95-1	1385.10	96	-	0.11	-	-	-	-	-	-	-	-	-	-	-	-	0.05	-	-	-	-
Fenchone	1195-79-5	1387.70	53	-	-	-	-	-	-	-	-	-	-	-	0.06	-	-	-	-	-	-	-
2-Methyl-4-Hydroxyacetophenone	875-59-2	N/A	80	-	-	-	-	-	0.30	-	-	-	-	-	-	-	-	-	-	-	-	-
Thymol	89-83-8	1403.00	83	-	-	-	-	-	-	-	-	-	-	-	-	-	-	-	-	-	-	-
Durenol	527-35-5	N/A	76	-	-	-	-	-	-	-	-	-	-	-	-	-	-	-	-	-	-	-
Rosefuran	15186-51-3	1403.50	97	-	0.40	0.22	0.08	0.09	-	-	-	-	-	-	-	8.05	-	0.09	0.06	0.05	0.09	-
3-Tert-Butylphenol	585-34-2	N/A	83	-	-	-	-	-	-	-	-	-	-	-	-	0.06	-	-	-	-	-	-
Perillen	539-52-6	1416.90	93	-	0.06	-	-	-	-	-	-	-	-	1.20	0.06	9.29	-	-	-	-	-	-
o-Methyl Anisole	578-58-5	1405.60	95	-	-	-	-	-	-	-	-	-	-	-	-	-	-	-	-	-	-	-
p-α-Dimethyl Styrene	1195-32-0	1432.90	96	-	-	-	0.14	-	-	-	-	-	-	-	0.07	0.09	-	-	-	-	-	-
(E)-Linalool Oxide (furanoid)	5989-33-3	1441.90	91	-	-	-	-	-	-	-	-	-	-	-	-	-	-	-	-	-	-	-
Cosmene	460-01-5	1444.60	96	-	0.32	0.27	-	0.19	-	-	-	-	-	-	-	0.12	0.16	0.12	0.14	-	-	-
Nerol Oxide	1786-08-9	N/A	96	-	-	-	-	-	-	-	-	-	-	-	-	0.40	-	-	-	-	-	-
(-)-limonene oxide	32543-51-4	1452.20	97	-	-	-	-	-	-	-	-	-	-	-	0.09	-	-	-	-	-	-	-
β-Terpineol	7299-41-4	N/A	95	-	-	-	-	-	-	-	-	-	-	-	0.51	-	-	-	-	-	-	-

α-Cubebene	17699-14-8	1459.50	98	-	0.14	-	-	0.11	-	-	0.66	0.48	0.09	-	-	-	-	0.22	-	-	0.77	-
Ethyl 2-(5-methyl-5-vinyltetrahydrofuran-2-yl)propan-2-yl carbonate	1000373-80-3	1468.60	95	0.25	0.49	0.86	0.97	0.14	5.29	0.52	-	0.26	-	0.17	-	-	0.65	-	-	-	-	-
(+)-Cyclosativene	22469-52-9	1479.10	99	-	-	-	-	-	-	-	-	-	-	-	-	-	-	0.06	-	-	-	-
Cyperene	2387-78-2	1479.60	98	-	-	-	-	1.84	-	-	-	-	-	-	-	-	-	-	-	-	-	-
Copaene	3856-25-5	1485.10	99	-	0.67	-	-	6.81	-	0.22	4.11	2.08	0.54	-	-	-	-	1.09	5.46	-	-	0.14
Theaspirane	36431-72-8	1491.30	86	-	-	-	-	-	-	-	-	-	-	-	-	-	-	-	-	0.15	-	0.09
(Z)-2-methylbutanal oxime	49805-56-3	1496.90	94	-	-	-	-	-	-	-	-	-	-	0.13	0.35	-	0.06	-	-	-	-	-
(E)-2-Methylbutanal Oxime	49805-55-2	1515.90	91	-	-	-	-	-	-	-	-	-	-	-	0.13	-	-	-	-	-	-	-
Pentadecane	629-62-9	N/A	93	-	-	-	-	-	-	-	-	-	-	-	-	0.05	-	-	-	-	-	-
Eicosane	112-95-8	N/A	78	-	-	-	-	-	-	-	-	-	-	-	-	0.16	-	-	-	-	-	-
2-Methyl-1-Pentene	763-29-1	N/A	58	-	-	-	-	-	-	-	-	-	-	-	-	-	-	-	-	-	0.85	-
β-Panasinsene	1000159-39-0	N/A	50	-	-	-	-	-	-	-	-	-	-	-	-	-	-	-	-	-	-	-
Cis-4(15),5-Muuroladiene	157477-72-0	1542.90	96	-	-	-	-	-	-	-	-	-	-	-	-	-	-	-	-	-	-	-
3-Methoxy-5-Methylphenol	3209-13-0	N/A	36	-	-	-	-	-	-	-	-	-	-	-	-	-	-	-	-	-	-	-
α-Bergamotene	17699-05-7	1563.90	96	-	-	-	-	-	-	-	-	-	-	-	0.11	-	-	-	-	-	-	-
β-Cubebene	13744-15-5	N/A	97	-	-	-	-	-	-	-	-	-	-	-	-	-	-	-	-	-	-	-
Linalool	78-70-6	1569.00	97	77.41	34.46	64.62	42.47	33.93	58.56	71.57	0.65	0.43	9.03	66.77	6.46	23.65	61.83	58.44	71.44	70.16	57.19	77.56
(E)-α-Bergamotene	13474-59-4	1577.30	98	-	-	-	-	0.06	-	-	-	-	0.12	-	0.08	-	-	-	-	-	-	-
β-Elemene	515-13-9	1589.80	91	-	0.30	-	-	0.38	-	0.13	2.65	0.44	-	-	-	-	-	0.45	0.27	-	0.75	0.07
β-Copaene	18252-44-3	1595.60	97	-	-	-	-	-	-	-	-	-	-	-	-	-	-	-	-	-	-	-
8,9-Dehydrothymol	18612-99-2	N/A	89	-	-	-	-	-	-	-	-	-	-	-	-	0.12	-	-	-	-	-	-
β-Caryophyllene	87-44-5	1600.40	99	-	0.22	-	-	0.17	-	0.08	1.74	0.38	-	-	-	-	-	0.33	-	-	0.45	-
4-Carvomenthenol	562-74-3	1603.40	97	-	-	-	-	-	-	-	-	-	-	-	0.34	0.08	-	-	-	-	-	-
(-)-Terpinen-4-ol	20126-76-5	1606.80	96	-	-	-	0.53	-	-	-	-	-	0.41	-	-	-	-	-	-	-	-	-
Dehydrolinalool	29957-43-5	1615.80	78	-	-	0.07	-	-	-	-	-	-	-	0.05	-	-	0.11	-	-	0.13	-	0.07
γ-Butyrolactone	96-48-0	N/A	49	-	-	-	-	-	-	-	-	-	-	-	-	-	-	-	-	-	-	-
β-Cyclocitral	432-25-7	1620.60	96	-	-	-	-	-	1.15	-	-	-	-	-	-	-	-	-	-	-	-	0.18
δ-Damascone	41436-42-4	N/A	70	-	-	-	-	-	-	-	-	-	-	-	-	-	-	-	-	-	-	-

(E)-α-Damascone	24720-09-0	1633.10	52	-	-	-	-	-	-	-	-	-	-	-	-	-	-	-	-	-	-	-
Naphthalene, 1,2,3,4,4a,5,6,7-octahydro-4-methyl-7-methylene-1-(1-methylethyl)-, (1S,4R,4aS)-	54274-73-6	1636.60	96	-	-	-	-	-	-	-	-	-	-	-	-	-	-	0.07	-	-	0.14	-
Rotundene	65128-08-7	1637.10	62	-	-	-	-	0.24	-	-	-	-	-	-	-	-	-	-	0.22	-	-	-
α-Damascone	31089-90-4	1637.50	58	-	-	-	-	-	-	-	-	-	-	-	-	-	-	-	-	-	-	-
(E)-β-Farnesene	18794-84-8	1641.50	38	-	-	-	-	-	-	-	-	-	-	-	-	-	-	-	-	-	0.05	-
2-epi-Trans-β-Caryophyllene	68832-35-9	1644.00	99	-	-	-	-	-	-	-	-	-	-	-	-	-	-	-	-	-	-	-
Safranal	116-26-7	1646.10	98	-	-	-	-	-	-	-	-	-	-	-	-	-	-	-	-	-	-	-
(Z)-Citral	106-26-3	N/A	96	-	-	-	-	-	-	-	-	-	-	-	-	2.18	-	-	-	-	-	-
(+)-γ-Gurjunene	22567-17-5	1653.70	90	-	-	-	-	-	-	-	-	-	-	-	-	-	-	-	-	-	-	-
10-Epizonarene	41702-63-0	1671.60	91	-	-	-	-	-	-	-	-	-	-	-	-	-	-	0.05	-	-	-	-
(-)-α-Gurjunene	489-40-7	1672.40	99	-	0.14	-	-	-	-	-	0.84	0.43	-	-	-	-	-	0.26	-	-	0.28	-
Piperitenone	491-09-8	1728.20	53	-	-	-	-	-	-	-	-	-	-	-	-	-	-	-	-	-	0.05	-
Eudesma-3,7(11)-Diene	6813-21-4	1742.10	58	-	-	-	-	0.17	-	-	-	-	-	-	-	-	-	-	-	-	-	-
δ-Selinene	473-14-3	N/A	93	-	-	-	-	-	-	-	-	-	-	-	-	-	-	-	-	-	-	-
β-Maaliene	489-29-2	N/A	93	-	-	-	-	-	-	-	-	-	-	-	-	-	-	-	0.24	-	-	-
(-)-Isoledene	95910-36-4	N/A	95	-	-	-	-	-	-	-	-	-	-	-	-	-	-	-	-	0.05	-	0.15
(+)-Calarene	17334-55-3	N/A	80	-	-	-	-	-	-	-	-	-	-	-	-	-	-	-	-	-	-	-
α-Panasinsene	56633-28-4	N/A	93	-	-	-	-	-	-	-	-	-	-	-	-	-	-	-	0.15	-	-	-
Alloaromadendrene	25246-27-9	N/A	53	-	-	-	-	-	-	-	-	-	-	-	-	-	-	-	-	-	0.05	-
γ-Muurolene	30021-74-0	1687.20	97	-	-	-	-	0.10	-	-	-	-	-	-	-	-	-	-	0.24	-	-	-
Ketoisophorone	1125-21-9	1693.90	91	-	-	-	-	-	-	-	-	-	-	-	-	-	-	-	-	-	-	-
Citral	5392-40-5	N/A	91	-	-	-	-	-	-	-	-	-	-	-	-	2.67	-	-	-	-	-	-
α-Terpineol	98-55-5	1699.00	91	0.13	0.46	-	-	0.44	0.66	0.30	-	0.54	20.07	2.60	31.54	2.25	0.23	0.52	0.26	0.74	0.43	0.53
(-)-α-Terpineol	10482-56-1	N/A	90	-	-	0.40	13.87	-	-	-	-	-	-	-	-	-	0.25	-	-	-	-	-
Valencene	4630-07-3	1706.00	96	-	-	-	-	-	-	-	-	-	-	-	-	-	-	-	-	-	0.22	-
(E)-Germacrene D	23986-74-5	1707.00	97	-	-	-	-	-	-	-	-	-	-	-	-	-	-	-	0.09	-	-	-
Eremophilene	10219-75-7	1709.50	96	-	0.10	-	-	-	-	-	0.86	-	-	-	-	-	-	0.14	-	-	-	-
Guaiene	88-84-6	N/A	94	-	-	-	-	-	-	-	-	-	-	-	-	-	-	-	-	-	-	-
α-Bulnesene	3691-11-0	1714.10	99	-	-	-	-	0.36	-	-	-	-	-	-	-	-	-	-	0.37	-	-	-

Longifolene-(V4)	61262-67-7	N/A	95	-	-	-	-	-	-	-	-	-	-	-	-	-	-	-	-	-	-	-
Guaia-9,11-diene	1000374-19-8	N/A	94	-	-	-	-	-	-	-	-	-	-	-	-	-	-	-	-	-	-	-
β-Isocomene	71596-72-0	N/A	93	-	-	-	-	-	-	-	-	-	-	-	-	-	-	-	-	-	-	-
Geranyl Acetate	105-87-3	N/A	91	-	-	-	-	-	-	-	-	-	-	-	-	0.19	-	-	-	-	-	-
α-Selinene	473-13-2	1727.00	96	-	3.23	-	-	-	-	1.61	23.21	1.87	2.67	-	-	-	-	-	-	0.27	4.89	0.57
Selina-4(15),7(11)-Diene	515-17-3	N/A	96	-	0.18	-	-	1.20	-	-	-	-	-	-	-	-	-	1.88	0.45	-	0.36	-
3,5,5-Trimethyl-4-Methylene-2-Cyclohexenone	20548-00-9	1729.20	90	-	-	-	-	-	-	-	-	-	-	-	-	-	-	-	-	-	-	-
α-Phellandren-8-ol	1686-20-0	1730.00	47	-	-	-	-	-	-	-	-	-	-	-	0.06	-	-	-	-	-	-	-
γ-Maalinene	20071-49-2	N/A	94	-	-	-	-	-	-	-	-	-	-	-	-	-	-	-	-	-	-	-
2-Acetyl-3,5-Dimethyl Pyrazine	54300-08-2	N/A	43	-	-	-	-	-	-	-	-	-	-	-	-	-	-	-	-	-	-	-
D-Citronellol	1117-61-9	N/A	96	-	-	-	-	-	-	-	-	-	-	-	-	0.28	-	-	-	-	-	-
(E)-linalool oxide (pyranoid)	39028-58-5	1742.10	72	-	-	0.15	0.11	-	0.55	-	-	-	-	-	-	-	0.06	-	-	0.4	-	0.41
δ-Cadinene	483-76-1	N/A	98	-	-	-	-	-	-	-	0.96	-	-	-	-	-	-	0.06	0.19	-	-	-
(+)-δ-Amorphene	16729-01-4	1757.00	97	-	-	-	-	0.15	-	-	-	-	-	-	-	-	-	-	-	-	-	-
1,5,9-Trimethyl-1,5,9-Cyclododecatriene	21064-19-7	N/A	64	-	-	-	-	-	-	-	-	-	-	-	-	-	-	-	-	-	-	-
β-Selinene	17066-67-0	1761.50	99	-	0.09	-	-	-	-	-	0.90	-	-	-	-	-	-	0.1	-	-	0.12	-
Myrtenol	515-00-4	N/A	87	-	-	-	-	-	-	-	-	-	-	-	0.05	-	-	-	-	-	-	-
Nerol	106-25-2	1804.80	96	-	-	0.07	-	-	-	-	-	-	-	0.14	-	18.50	0.12	-	-	-	0.09	-
Dihydro-β-Ionone	17283-81-7	1833.50	99	-	-	-	-	-	-	-	-	-	-	-	-	-	-	-	0.09	0.24	0.32	1.53
Geraniol	106-24-1	1852.70	97	0.19	-	0.19	-	-	-	-	-	-	-	0.13	-	4.62	-	-	-	-	-	-
Phenylethanol	60-12-8	1921.20	91	-	-	-	-	-	-	-	-	-	-	-	-	-	0.05	-	-	-	-	-
(E)-β-Ionone	79-77-6	1940.60	96	-	-	-	-	-	-	-	-	-	-	-	-	-	-	-	-	-	-	-
Cubebol	23445-02-5	N/A	89	-	-	-	-	-	-	-	-	-	-	-	-	-	-	0.08	-	-	0.05	-
Dihydro-β-Ionol	3293-47-8	1971.50	99	-	-	-	-	-	-	-	-	-	-	-	-	-	-	-	-	-	-	0.15
Laureth-7	3055-97-8	N/A	72	-	-	-	-	-	-	-	-	-	-	0.68	-	0.33	-	-	-	-	-	-

Compounds	CAS	RI	SI (%)	Relative content (%)														
				GRU	RR	TW	CH	DXQ	DH	FT	WR-HJ-01	RR-RX-01	CA-WR-01	HTG-RR-02	RR-DH-03	GR-RP-02	GR-RP-05	GR-RR-06
Ethyl DL-Leucate	10348-47-7	N/A	9	-	-	-	-	-	-	-	-	-	-	-	-	-	-	-
Vinyl Formate	692-45-5	N/A	5	-	-	-	-	-	-	-	-	-	-	-	-	-	-	-
Acetaldehyde	75-07-0	N/A	83	-	-	-	-	-	-	-	-	-	-	-	-	-	0.82	-
4-Methyl-1,3-Pentadiene	926-56-7	N/A	94	-	-	-	-	-	-	-	-	-	-	-	-	-	-	-
3-Methylfuran	930-27-8	941.82	91	-	-	-	-	-	-	-	-	-	-	-	-	-	-	-
2-Methyl Furan	534-22-5	941.97	91	-	-	-	-	-	-	-	-	-	-	-	-	-	-	-
Methyl ethyl ketone	78-93-3	943.87	86	-	-	-	-	-	-	-	-	-	-	-	-	-	-	-
Ethyl Acetate	141-78-6	N/A	91	-	-	-	-	0.47	-	-	-	-	-	0.05	-	-	-	-
Nonane	111-84-2	N/A	86	-	-	-	-	-	-	-	-	-	-	-	-	0.13	-	-
Ethanol	64-17-5	959.32	91	-	-	1.03	-	-	-	-	0.79	1.34	-	17.92	-	5.56	0.51	0.23
α-Thujene	2867-05-2	1021.30	94	-	-	-	-	-	-	-	-	-	-	-	-	-	-	-
Toluene	108-88-3	1035.70	74	-	-	-	-	-	-	-	-	-	-	-	-	-	-	-
L-α-Pinene	7785-26-4	N/A	97	-	-	-	-	-	-	-	0.64	-	-	0.22	0.28	-	-	-
Cyclofenchene	488-97-1	N/A	97	-	-	-	-	-	-	-	-	-	-	-	-	-	-	-
(+)-α-Pinene	7785-70-8	1015.90	97	-	0.22	-	1.81	-	0.22	0.07	-	0.07	-	0.12	-	0.16	-	-
Camphene	79-92-5	1056.10	97	-	-	-	-	-	-	-	-	-	-	-	-	-	-	-
2-Methyl Butyronitrile	18936-17-9	1085.70	59	-	-	-	-	-	-	-	-	-	-	0.12	-	0.23	-	-
Undecane	1120-21-4	1091.10	95	0.06	-	-	-	-	-	-	-	-	-	-	0.07	0.08	-	0.05
(-)-β-Pinene	18172-67-3	1091.50	94	-	-	-	-	-	-	-	-	-	-	-	-	-	-	-
(-)-Camphene	5794-04-7	N/A	97	-	-	-	0.05	-	-	-	0.32	-	-	-	-	-	-	-
1,4-Butanediol Diacrylate	1070-70-8	N/A	56	-	0.07	-	-	-	-	-	-	-	-	-	-	-	-	-
Phytane	638-36-8	N/A	72	-	-	-	-	-	0.12	-	-	-	-	-	-	-	-	-
2-Methyl Decane	6975-98-0	N/A	50	-	0.08	-	-	-	-	-	-	-	-	-	-	-	-	-
β-Pinene	127-91-3	N/A	90	-	-	-	0.47	-	0.15	-	0.22	-	-	-	-	-	-	-
β-Thujene	28634-89-1	1105.90	94	-	-	-	0.14	-	-	-	0.05	-	-	-	-	-	-	-
Sabinene	3387-41-5	1107.20	96	0.05	-	-	1.76	-	-	-	-	-	-	-	0.21	0.14	-	-
α-Phellandrene	99-83-2	1142.70	91	-	-	-	-	-	-	-	-	-	-	-	-	-	-	-
Agmatine	306-60-5	1143.40	17	-	-	-	-	-	-	-	-	-	-	-	-	0.21	-	-
β-Myrcene	123-35-3	1148.80	93	0.78	0.69	0.64	3.16	0.51	0.79	0.77	0.99	0.64	0.9	0.37	1.5	1.79	1.04	1.05
(+)-2-Carene	4497-92-1	N/A	97	-	0.06	-	-	-	-	-	-	-	-	-	-	-	-	-

1-Methylene-2-Methyl-3-Isopropenylcyclopentane	56710-83-9	N/A	91	-	-	-	-	-	-	-	-	-	-	-	-	0.12	-	-
α-Terpinene	99-86-5	1156.50	97	-	-	-	-	-	-	-	-	-	-	-	-	-	-	-
2,3-Dihydro-1,8-Cineole	92760-25-3	1182.70	72	-	-	-	0.07	-	-	-	-	-	-	-	-	-	-	-
Eucalyptol	470-82-6	1191.10	90	-	-	-	2.95	-	0.34	0.05	-	-	-	0.13	0.25	0.08	-	-
Dipentene	138-86-3	1193.20	94	-	-	-	-	-	-	-	-	-	-	-	-	-	-	-
(E)-dehydroxylinalool oxide	54750-70-8	N/A	97	-	0.14	-	-	-	-	0.08	0.05	0.08	-	-	0.06	-	0.11	0.11
D-Limonene	5989-27-5	1176.10	97	0.2	0.77	0.12	5.93	0.3	0.83	0.3	1.19	0.27	0.39	0.77	1.3	1.18	0.33	0.4
(Z)-Dehydroxylinalool Oxide	54750-69-5	1198.90	59	-	-	-	-	-	-	-	-	-	-	-	-	0.16	-	-
β-Terpinene	99-84-3	1219.70	91	-	-	-	-	-	-	-	-	-	-	-	-	-	-	-
(E)-β-Ocimene	13877-91-3	1241.00	94	49.7	1.63	4.74	0.54	2.35	0.08	39.44	1.86	6.58	0.89	8.53	10.44	14.01	29.22	16.16
3,7,7-trimethyl-1,3,5-cycloheptatriene	3479-89-8	N/A	81	-	-	-	-	-	-	-	-	-	-	-	-	-	-	-
(1S,3S)-(E)-4-Carene	5208-50-4	1261.40	91	-	-	-	-	0.1	-	-	-	-	-	-	-	-	-	-
(+)-3-Carene	498-15-7	N/A	94	-	-	-	-	-	-	-	-	0.05	-	-	-	-	-	-
α-Pinene	80-56-8	N/A	95	-	-	-	-	-	-	-	-	-	-	-	0.25	-	-	-
β-phellandrene	555-10-2	1184.30	95	-	-	-	-	-	0.16	-	-	-	-	-	-	-	-	-
γ-Terpinene	99-85-4	1226.90	97	-	-	-	0.65	0.05	0.12	-	-	-	-	-	-	0.25	-	-
(Z)-β-Ocimene	3338-55-4	1239.60	95	-	4.93	52.69	0.55	-	0.47	-	-	26.14	1.48	-	-	-	-	-
α-Ocimene	502-99-8	1243.50	95	-	-	-	-	-	0.1	-	-	-	-	-	-	-	-	-
Cyclopropane, 1,1-dimethyl-2-(3-methyl-1,3-butadien-1-yl)-	68998-21-0	N/A	93	-	-	-	-	-	-	0.1	-	-	-	-	-	-	-	0.05
Bicyclo[3.1.1]hept-2-ene,3	4889-83-2	1254.60	94	-	-	-	-	-	-	-	-	-	-	0.44	-	-	-	-
o-Cymene	527-84-4	1256.90	94	-	-	-	-	-	0.06	-	-	-	-	-	-	-	0.05	-
Isoterpinolene	586-63-0	1259.40	90	-	-	-	-	-	-	-	-	-	-	-	-	-	-	-
m-Cymene	535-77-3	1263.50	93	-	-	-	0.06	-	-	-	-	-	-	-	0.12	0.12	-	-
Terpinolene	586-62-9	1266.20	97	-	-	-	0.65	0.12	0.11	0.06	0.14	0.07	0.05	0.1	0.15	0.51	0.11	0.21
(+)-4-Carene	29050-33-7	1267.40	96	-	-	-	0.17	-	0.08	-	-	-	-	0.09	-	-	-	-
p-Cymene	99-87-6	1270.40	91	-	-	-	0.08	-	-	-	-	-	-	0.16	-	-	-	-
δ-3-carene	13466-78-9	N/A	91	0.13	0.14	-	-	-	-	-	-	-	-	-	0.06	-	-	-

2-Ethyl-1,4-Dimethyl Benzene	1758-88-9	N/A	60	-	-	0.07	-	-	-	-	-	-	-	0.11	0.06	-	-	-
2-Carene	554-61-0	N/A	96	-	-	0.05	-	-	-	-	-	-	-	-	-	-	-	-
Tridecane	629-50-5	1295.20	95	-	-	-	-	-	-	-	-	-	-	-	-	-	-	-
Oxalic acid, allyl pentyl ester	1000309-23-2	1332.10	64	-	-	-	-	-	-	-	-	-	-	-	-	0.51	-	-
Methyl Heptenone	110-93-0	1336.30	92	-	-	-	-	-	-	-	-	-	-	-	-	-	-	-
Pentyl Propyl Ether	18641-82-2	N/A	78	-	-	-	-	-	-	-	-	-	-	-	0.09	-	-	-
Alloocimene	673-84-7	1372.60	94	0.59	0.05	0.67	0.06	0.1	-	0.44	0.06	0.41	0.05	-	0.07	0.26	0.38	0.26
1,5,5,6-Tetramethylcyclohexa-1,3-Diene	514-94-3	1375.90	94	-	-	-	-	-	-	-	-	-	-	0.06	-	-	-	-
3,4-Dimethyl-2,4,6-Octatriene	57396-75-5	1378.60	87	-	0.09	-	0.22	-	-	-	-	-	-	0.08	-	-	-	-
1,2,3,4,5-Pentamethylcyclopentadiene	4045-44-7	1381.30	97	-	0.05	-	-	-	-	-	-	-	-	-	-	-	-	-
p-Mentha-1,3,8-Triene	18368-95-1	1385.10	96	0.22	-	0.39	-	-	-	0.16	-	0.26	0.08	0.1	-	-	0.07	-
Fenchone	1195-79-5	1387.70	53	-	-	-	-	-	-	-	-	-	-	-	-	-	-	-
2-Methyl-4-Hydroxyacetophenone	875-59-2	N/A	80	-	-	-	-	-	-	-	-	-	-	-	-	-	-	-
Thymol	89-83-8	1403.00	83	-	-	-	0.28	-	-	-	-	-	-	-	-	-	-	-
Durenol	527-35-5	N/A	76	-	0.24	-	-	-	-	-	-	0.38	-	-	-	-	-	-
Rosefuran	15186-51-3	1403.50	97	0.56	0.13	1.08	-	0.06	-	0.4	-	0.57	-	0.39	0.23	0.53	1.15	0.77
3-Tert-Butylphenol	585-34-2	N/A	83	-	-	0.45	-	-	-	-	-	-	-	-	-	-	-	-
Perillen	539-52-6	1416.90	93	-	0.14	-	-	0.06	-	-	-	0.05	-	-	-	0.07	0.06	0.08
o-Methyl Anisole	578-58-5	1405.60	95	-	-	-	-	-	-	-	-	-	-	-	0.06	-	-	-
p-α-Dimethyl Styrene	1195-32-0	1432.90	96	-	-	-	-	-	-	-	-	-	-	-	-	-	-	-
(E)-Linalool Oxide (furanoid)	5989-33-3	1441.90	91	-	-	-	-	-	0.13	-	-	-	-	-	-	-	-	-
Cosmene	460-01-5	1444.60	96	0.05	0.19	0.29	-	0.17	-	0.05	0.15	0.15	-	-	-	0.2	-	-
Nerol Oxide	1786-08-9	N/A	96	-	-	-	-	-	-	-	-	-	-	-	-	-	-	-
(-)-limonene oxide	32543-51-4	1452.20	97	-	-	-	-	-	-	-	-	-	-	-	-	-	-	-
β-Terpineol	7299-41-4	N/A	95	-	-	-	-	-	-	-	-	-	-	-	-	-	-	-

α-Cubebene	17699-14-8	1459.50	98	-	-	0.46	0.06	0.26	-	0.57	0.05	0.15	1.45	-	-	1.49	-	-
Ethyl 2-(5-methyl-5-vinyltetrahydrofuran-2-yl)propan-2-yl carbonate	1000373-80-3	1468.60	95	-	-	-	-	-	-	-	0.56	0.79	0.41	-	1.63	0.84	2.02	2.78
(+)-Cyclosativene	22469-52-9	1479.10	99	-	-	-	-	0.08	-	-	0.06	0.08	-	-	-	0.12	-	-
Cyperene	2387-78-2	1479.60	98	-	-	1.39	-	0.12	-	-	1.98	1.75	0.07	-	-	-	-	-
Copaene	3856-25-5	1485.10	99	0.09	-	4.89	-	2.57	-	0.07	8.26	7.13	-	-	-	-	0.14	-
Theaspirane	36431-72-8	1491.30	86	-	0.19	-	-	-	-	-	-	-	-	0.19	-	0.13	-	-
(Z)-2-methylbutanal oxime	49805-56-3	1496.90	94	-	-	-	-	-	-	-	-	-	-	-	-	-	-	-
(E)-2-Methylbutanal Oxime	49805-55-2	1515.90	91	-	-	-	-	-	-	-	-	-	-	-	-	-	-	-
Pentadecane	629-62-9	N/A	93	-	-	-	-	-	-	-	-	-	-	-	-	-	-	-
Eicosane	112-95-8	N/A	78	-	-	-	-	-	-	-	-	-	-	-	-	-	-	-
2-Methyl-1-Pentene	763-29-1	N/A	58	-	-	-	-	-	-	-	-	-	-	-	-	-	-	-
β-Panasinsene	1000159-39-0	N/A	50	-	-	-	-	-	-	-	-	0.16	-	-	-	-	-	-
Cis-4(15),5-Muuroladiene	157477-72-0	1542.90	96	-	-	-	-	-	-	-	-	-	-	-	-	0.07	-	-
3-Methoxy-5-Methylphenol	3209-13-0	N/A	36	-	0.06	-	-	-	-	-	-	-	-	-	-	-	-	-
α-Bergamotene	17699-05-7	1563.90	96	-	-	-	-	-	-	-	-	-	-	-	-	-	-	-
β-Cubebene	13744-15-5	N/A	97	-	-	-	-	-	-	-	0.06	0.12	-	-	-	-	-	-
Linalool	78-70-6	1569.00	97	38.85	21.55	23.18	58.49	59.72	71.78	42.72	70.39	41.22	82.86	6.03	60.97	33.38	55.53	69.11
(E)-α-Bergamotene	13474-59-4	1577.30	98	-	-	-	0.05	-	-	-	-	-	-	-	-	-	-	-
β-Elemene	515-13-9	1589.80	91	-	-	0.19	-	0.76	-	0.47	0.31	0.54	0.2	-	-	0.26	-	-
β-Copaene	18252-44-3	1595.60	97	-	-	0.07	-	-	-	-	-	-	-	-	-	-	-	-
8,9-Dehydrothymol	18612-99-2	N/A	89	-	-	-	-	-	-	-	-	-	-	-	-	-	-	-
β-Caryophyllene	87-44-5	1600.40	99	-	-	-	-	0.38	-	0.2	0.05	0.16	0.12	-	-	0.41	-	-
4-Carvomenthenol	562-74-3	1603.40	97	-	-	-	0.31	-	0.06	-	-	-	-	-	-	-	-	-
(-)-Terpinen-4-ol	20126-76-5	1606.80	96	-	-	-	-	-	-	-	-	-	-	-	0.05	-	-	-
Dehydrolinalool	29957-43-5	1615.80	78	-	-	-	-	-	-	-	-	0.06	0.06	-	-	-	0.11	0.13
γ-Butyrolactone	96-48-0	N/A	49	-	-	-	-	-	-	-	-	-	-	0.07	-	-	-	-
β-Cyclocitral	432-25-7	1620.60	96	-	-	-	0.45	-	0.21	-	-	-	-	-	-	-	-	-
δ-Damascone	41436-42-4	N/A	70	-	-	-	-	-	0.05	-	-	-	-	-	-	-	-	-

(E)-α-Damascone	24720-09-0	1633.10	52	-	-	-	-	-	-	-	-	-	-	0.08	-	-	-	-
Naphthalene, 1,2,3,4,4a,5,6,7-octahydro-4-methyl-7-methylene-1-(1-methylethyl)-, (1S,4R,4aS)-	54274-73-6	1636.60	96	-	-	-	-	0.1	-	0.05	-	-	-	-	-	-	-	-
Rotundene	65128-08-7	1637.10	62	-	-	0.23	-	0.09	-	-	0.31	0.24	0.05	-	-	-	-	-
α-Damascone	31089-90-4	1637.50	58	-	0.1	-	-	-	-	-	-	-	-	-	-	-	-	-
(E)-β-Farnesene	18794-84-8	1641.50	38	-	-	-	-	-	-	-	-	-	-	-	-	-	-	-
2-epi-Trans-β-Caryophyllene	68832-35-9	1644.00	99	-	-	-	-	-	-	-	0.06	-	-	-	-	-	-	-
Safranal	116-26-7	1646.10	98	-	-	-	-	-	0.1	-	-	-	-	-	-	-	-	-
(Z)-Citral	106-26-3	N/A	96	-	-	-	-	-	-	-	-	-	-	-	-	-	-	-
(+)-γ-Gurjunene	22567-17-5	1653.70	90	-	-	-	-	0.11	-	-	0.06	0.05	-	-	-	-	-	-
10-Epizonarene	41702-63-0	1671.60	91	-	0.07	-	-	0.11	-	-	-	-	-	0.19	-	0.11	-	-
(-)-α-Gurjunene	489-40-7	1672.40	99	-	-	-	-	0.31	-	0.14	0.11	-	0.12	-	-	0.35	-	-
Piperitenone	491-09-8	1728.20	53	-	-	-	-	-	-	-	-	-	-	-	-	-	-	-
Eudesma-3,7(11)-Diene	6813-21-4	1742.10	58	-	-	-	-	-	-	-	-	-	-	-	-	-	-	-
δ-Selinene	473-14-3	N/A	93	-	-	-	-	-	-	-	-	-	0.1	-	-	-	-	-
β-Maaliene	489-29-2	N/A	93	-	-	0.21	-	0.15	-	-	0.26	0.21	-	-	-	-	-	-
(-)-Isoledene	95910-36-4	N/A	95	-	-	-	-	-	-	0.06	-	-	0.05	-	-	-	-	-
(+)-Calarene	17334-55-3	N/A	80	-	-	0.11	-	-	-	-	-	-	-	-	-	-	-	-
α-Panasinsene	56633-28-4	N/A	93	-	-	-	-	-	-	-	-	-	-	-	-	-	-	-
Alloaromadendrene	25246-27-9	N/A	53	-	-	-	-	-	-	-	-	-	-	-	-	-	-	-
γ-Muurolene	30021-74-0	1687.20	97	-	-	0.11	-	0.1	-	-	0.13	0.12	-	-	-	-	-	-
Ketoisophorone	1125-21-9	1693.90	91	-	-	-	0.07	-	0.06	-	-	-	-	-	-	-	-	-
Citral	5392-40-5	N/A	91	-	-	-	-	-	-	-	-	-	-	-	-	-	-	-
α-Terpineol	98-55-5	1699.00	91	0.18	0.4	0.11	9.71	0.25	-	0.46	0.27	0.37	0.33	0.36	1.6	0.74	0.41	-
(-)-α-Terpineol	10482-56-1	N/A	90	-	-	-	-	-	2.47	-	-	-	-	-	-	-	-	0.73
Valencene	4630-07-3	1706.00	96	-	-	-	-	-	-	-	-	-	-	-	-	-	-	-
(E)-Germacrene D	23986-74-5	1707.00	97	-	-	0.07	-	-	-	-	0.09	0.14	-	-	-	-	-	-
Eremophilene	10219-75-7	1709.50	96	-	-	-	-	0.22	-	0.13	-	-	0.06	-	-	-	-	-
Guaiene	88-84-6	N/A	94	-	-	-	-	-	-	-	-	-	-	-	-	0.06	-	-
α-Bulnesene	3691-11-0	1714.10	99	-	-	0.31	-	-	-	-	0.1	0.09	-	-	-	-	-	-

Longifolene-(V4)	61262-67-7	N/A	95	-	-	-	-	0.27	-	-	-	-	-	-	-	-	-	-
Guaia-9,11-diene	1000374-19-8	N/A	94	-	-	-	-	-	-	-	-	0.3	-	-	-	-	-	-
β-Isocomene	71596-72-0	N/A	93	-	-	-	-	-	-	-	0.34	-	-	-	-	-	-	-
Geranyl Acetate	105-87-3	N/A	91	-	-	-	-	-	-	-	-	-	-	-	-	-	-	-
α-Selinene	473-13-2	1727.00	96	-	0.16	-	0.53	-	-	2.88	-	-	0.13	0.08	0.27	-	0.27	0.14
Selina-4(15),7(11)-Diene	515-17-3	N/A	96	0.78	-	-	-	4.57	0.38	0.18	-	1.16	0.84	-	-	0.89	-	-
3,5,5-Trimethyl-4-Methylene-2-Cyclohexenone	20548-00-9	1729.20	90	-	-	-	-	-	-	-	0.14	-	-	-	-	-	-	-
α-Phellandren-8-ol	1686-20-0	1730.00	47	-	-	-	-	-	-	-	-	-	-	-	-	-	-	-
γ-Maalinene	20071-49-2	N/A	94	-	-	0.29	-	-	-	-	-	-	-	-	-	-	-	-
2-Acetyl-3,5-Dimethyl Pyrazine	54300-08-2	N/A	43	-	-	-	-	-	0.06	-	-	-	-	-	-	-	-	-
D-Citronellol	1117-61-9	N/A	96	-	-	-	-	-	-	-	-	-	-	-	-	-	-	-
(E)-linalool oxide (pyranoid)	39028-58-5	1742.10	72	-	0.07	-	0.09	-	0.11	-	-	0.27	-	-	0.28	0.16	0.2	0.31
δ-Cadinene	483-76-1	N/A	98	-	-	-	-	-	-	-	-	-	0.09	-	-	-	-	-
(+)-δ-Amorphene	16729-01-4	1757.00	97	-	-	0.21	-	0.18	-	-	0.24	0.22	-	-	-	0.19	-	-
1,5,9-Trimethyl-1,5,9-Cyclododecatriene	21064-19-7	N/A	64	-	-	-	-	-	-	-	0.2	-	0.1	-	-	-	-	-
β-Selinene	17066-67-0	1761.50	99	-	-	0.1	-	-	-	0.08	-	-	-	-	-	0.06	-	-
Myrtenol	515-00-4	N/A	87	-	-	-	-	-	-	-	-	-	-	-	-	-	-	-
Nerol	106-25-2	1804.80	96	-	-	-	-	-	-	-	-	-	-	-	-	-	-	-
Dihydro-β-Ionone	17283-81-7	1833.50	99	-	0.07	0.05	0.6	-	0.2	0.08	0.26	0.05	-	0.14	0.17	0.16	0.08	-
Geraniol	106-24-1	1852.70	97	-	-	-	-	-	-	-	-	-	-	-	-	-	-	-
Phenylethanol	60-12-8	1921.20	91	-	-	-	-	-	-	-	-	-	-	-	-	-	-	-
(E)-β-Ionone	79-77-6	1940.60	96	-	-	-	0.11	-	-	-	-	-	-	-	-	-	-	-
Cubebol	23445-02-5	N/A	89	-	-	-	-	-	-	-	-	-	-	-	-	-	-	-
Dihydro-β-Ionol	3293-47-8	1971.50	99	-	-	-	0.06	-	-	-	-	-	-	-	-	-	-	-
Laureth-7	3055-97-8	N/A	72	-	-	-	-	-	-	-	-	-	-	-	-	-	-	-
